# Temporal and quantitative variability in muscle electrical activity decreases as dexterous hand motor skills are learned

**DOI:** 10.1371/journal.pone.0236254

**Published:** 2020-07-20

**Authors:** Toshiyuki Aoyama, Yutaka Kohno

**Affiliations:** 1 Department of Physical Therapy, Ibaraki Prefectural University of Health Sciences, Inashiki-gun, Ibaraki, Japan; 2 Centre for Medical Sciences, Ibaraki Prefectural University of Health Sciences, Inashiki-gun, Ibaraki, Japan; Duke University, UNITED STATES

## Abstract

Muscle activity changes quantitatively and temporally during the motor learning process. However, the association between variability in muscle electrical activity and the learning and performance of dexterous hand movements is not well understood. Therefore, we undertook this study to investigate the relationships between temporal and quantitative variabilities in muscle activity and the learning of motor skills. Thirty-eight healthy participants performed 30 trials of a task that measured the time taken to rotate two cork balls 20 times using their non-dominant hand. The electromyographic (EMG) activities of the abductor pollicis brevis (APB), first dorsal interosseous, and extensor digitorum (ED) muscles were recorded. Temporal and quantitative variabilities in the EMG activity were evaluated by calculating the coefficient of variation of the duration and area of EMG activation. As motor learning proceeded, the task was completed more quickly and the EMG variability decreased. For all three muscles, significant correlations were observed between individual participants’ ball rotation time and EMG variability. Furthermore, significant positive correlations were observed between improvement in ball rotation time and reduction in EMG variability for the APB and ED muscles. These novel findings provide important insights regarding the relationships between temporal and quantitative variabilities in muscle activity and the learning of fine motor skills.

## Introduction

Efficient and coordinated muscle activity plays an essential role in fine motor skills. Quantitative changes in muscle electrical activity before and during movements occur in various motor learning tasks [1–7]. In most of these studies, the electromyography (EMG) amplitude decreases with motor learning [3–7]. With respect to the temporal aspect of the EMG changes, several studies have revealed that latency [2, 4, 7], time to peak EMG activity [2], and duration [3] of agonist EMG activity are reduced with motor learning. In addition, Bruecknera et al. have shown that practice-related EMG intensity shifts from higher to lower frequency bands in a dynamic balance learning task [6]. Recently, muscle synergy analysis has revealed that long-term motor training modifies the structure of the coordination patterns of muscles during walking and balancing [8]. These findings suggest that changes in quantitative, temporal, and coordinated patterns of muscle activity are closely related to motor skills or its learning.

For more than 30 years, studies have reported about the intra- and inter-individual variabilities in muscle electrical activity during cyclical movements, such as walking [9], running [10], and swimming [11]. A study that assessed changes in muscle electrical activity during the development of walking reported that the variability in EMG activity during walking was greater in healthy children than in adults [12]. A subsequent study demonstrated greater variability in EMG activity during walking in children aged 7–9 years than in those aged 13–16 years [13]. These findings suggest that variability in muscle electrical activity during walking decreases with growth. On the other hand, there are few reports on the relationship between variability in muscle electrical activity and motor skills or learning.

Two studies have reported that the variability in muscle activity decreases with simple learning tasks involving single-joint movements [14, 15]. However, it remains unclear whether variability in EMG activity is also reduced after acquiring dexterous hand motor skills, such as the ball rotation task [16, 17]. We speculated that the extent to which each muscle contributes to newly acquired dexterous motor skills might be reflected in the degree of change in its EMG variability. Therefore, the primary purpose of this study was to determine whether temporal and quantitative variability in EMG activity is reduced in the learning process of the ball rotation task, which requires the coordinated activities of several muscles, and whether the degree of reduction in EMG variability changes depending on the contribution of each muscle used for the task. Moreover, it is unclear whether individual differences in EMG variability account for individual differences in motor skills. If the variability in EMG activity is related to individual differences in motor skills, it may be useful as an index reflecting motor skills. Therefore, the secondary aim of this study was to investigate whether individual differences in the variability in EMG activity are related to individual dexterous hand motor skills.

## Methods

### Participants

The study included 38 neurologically healthy participants (mean ± SD age, 21.0 ± 1.9 years; 24 men and 14 women). All participants were right-handed according to the Edinburgh Handedness Inventory [18]. This study was performed in accordance with the recommendations of the Declaration of Helsinki established by the World Medical Association. The protocol was approved by the Ethics Committee of the Ibaraki Prefectural University of Health Sciences. All subjects gave written informed consent in accordance with the Declaration of Helsinki.

### Motor task

The motor task used in this study was the two-ball rotation task [16, 17]. Participants sat comfortably in a chair with their left forearm lying on a side table. Then, the participants were instructed to rotate two cork balls (diameter, 40 mm) 20 times counterclockwise using their left hand. According to the verbal instructions of the experimenter, the participants had to open their left hand and place the two cork balls back and forth on their left palm with their forearm on the table. They started this task after lighting of the LED. During this motor task, participants were instructed to not drop the ball while rotating it as quickly as possible. A stopwatch was used to record the ball rotation time (the time taken from LED lighting to the end of the task). The ball rotation time (the time taken to complete a trial) is the speed at which a trial was performed, and it is expected that the time would decrease with learning. If the participants dropped the ball, the trial was terminated at that time. This trial was defined as an error trial, and an additional trial was performed. The error trials were not included in the analysis. The participants practiced this motor task until they accomplished 30 successful trials. To reduce the effects of fatigue on the motor performance, participants were given a 30-s break between trials and a 5-min break after every 10 trials.

### EMG recording

Before attaching the EMG electrodes, the participant’s skin was rubbed with alcohol and abraded with an abrasive skin preparation gel. Custom-made Ag/AgCl bar electrodes (inter-electrode distance, 10 mm, Unique Medical, Japan) were placed over the abductor pollicis brevis (APB), first dorsal interosseous (FDI), and extensor digitorum (ED) muscles. We have selected the APB and ED muscles as the most relevant muscles for this motor task and the FDI muscle as a partly related muscle for the task. The EMG activity from other muscles was also recorded during the experiment but was not used for analysis herein. The EMG signals were amplified at a gain of 1000 using a Neuropack MEB-2300 system (Nihon Kohden, Japan) and band-pass filtered at 10–1000 Hz. The signals were sampled at 2000 Hz and stored in a laboratory computer for offline analysis. Muscle activity was first measured during the maximum voluntary contraction (MVC; 5 s isometric contraction, three times repetition) of each muscle and was then recorded throughout the ball rotation task.

### Analysis

For each participant, the average ball rotation time and number of errors in the initial stage (trials 1–5), middle stage (trials 13–17), and final stage (trials 26–30) of the ball rotation task were calculated.

**Fig 1:**
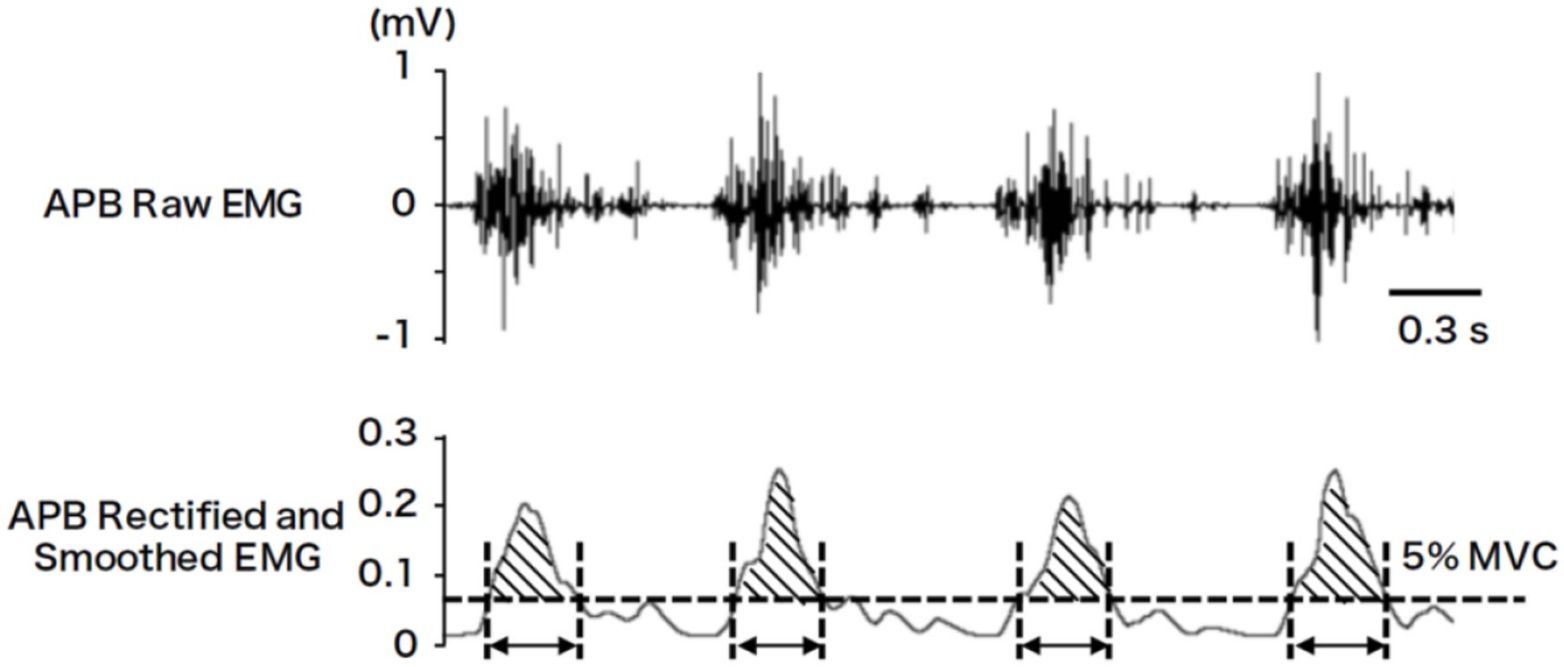
Analysis of electromyographic (EMG) activity. Abductor pollicis brevis (APB) muscle of a typical participant. The raw EMG trace for the APB shows phasic activity. The raw data were rectified and smoothed using a 5-Hz Butterworth low-pass filter. The phases during which muscle activity was ≥5% maximum voluntary contraction (MVC) were defined as on-phases. For each trial, the duration of each on-phase (within the limits of the two-way arrow) and the areas of the on-phases between the trace and the line representing the 5% MVC level (shaded area) were calculated.

Using a second—order digital Butterworth filter in both forward and backward direction in time, created using the LabVIEW 2017 software (National Instruments, USA), the EMG activities recorded from the APB, FDI, and ED muscles were band-pass filtered (20–500 Hz), full-wave rectified, and smoothed using a 5-Hz low-pass filter. The EMG signals were normalized by dividing them by the EMG activity measured in each muscle at the MVC. The criterion for the presence or absence of activity in a muscle was defined as 5% of the MVC for that muscle, with the period during which muscle activity exceeded this threshold, defined as the “on-phase”. The duration of the on-phases and the EMG area surrounded by the curve of the on-phases and 5% MVC line for each trial were measured (Fig 1). We used 5% MVC as a criterion because the total “on-phase” duration was approximately half of the ball rotation time in most participants and was therefore suitable for judging the presence or absence of muscle activity.

**Fig 2:**
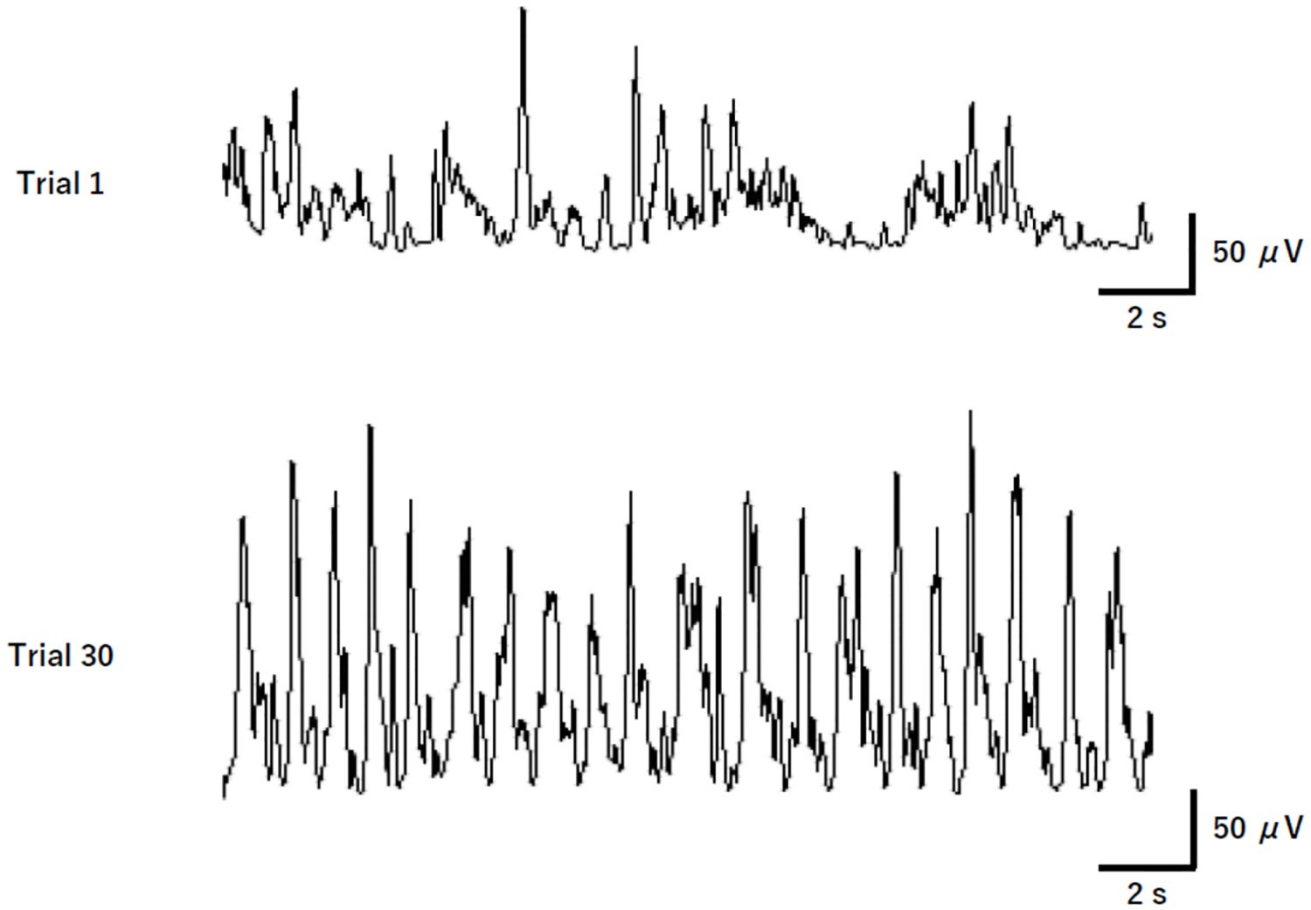
Typical rectified and smoothed electromyographic activity of the abductor pollicis brevis muscle. This figure shows that temporal and quantitative variabilities in muscle electrical activity are clearly reduced from the first (trial 1) to the final trial (trial 30).

To determine whether muscle activity decreases with task learning, as shown in many previous studies as well [3–7], we counted the number of EMG on-phases and calculated the total EMG area (sum of all EMG areas under the curve) and average EMG area for each trial. As shown in Fig 2, the EMG burst was not regular in the first trial; however, in the final trial, it was temporal, with enhancement in the quantitative reproducibility of the EMG bursts. Therefore, to investigate how temporal and quantitative variabilities in muscle electrical activity change during the process of motor learning, the coefficient of variation (CV) of both on-phase duration and on-phase area within a single trial was calculated. It was expected that these data, particularly in the muscles that are closely relevant to the task, decrease with learning. The number of on-phases, total and average EMG areas, and CV of on-phase duration and on-phase area were averaged for all the three stages. However, only a small number of EMG on-phases were obtained for the trial if there was strong muscle activity that persistently exceeded 5% MVC or if there was only weak muscle activity that never exceeded 5% MVC during the task. If the number of EMG on-phases was 0 or 1, the CV of on-phase duration or area could not be calculated. Therefore, the data for the relevant muscles of a participant for trials in which the number of EMG on-phases was 0 or 1 were not included in the analysis. As a result, the EMG activity of the APB, FDI, and ED muscles for one, one, and two participants, respectively, was excluded.

All statistical analyses were performed using SPSS version 22 statistical software (IBM, Armonk, NY, USA). The level of statistical significance was set as p < 0.05. We performed the Kolmogorov–Smirnov test to examine the null hypothesis stating that the obtained data were normally distributed. As a result, because the null hypothesis stating that the variables included in the number of EMG on-phases are normally distributed was not rejected, one-way repeated measure ANOVA and Bonferroni’s multiple comparisons were performed. For all the other statistical analysis, nonparametric tests were used since the null hypothesis of a normal distribution was rejected. Friedman’s and Wilcoxon signed-rank tests for post-hoc comparisons using Bonferroni’s correction were applied to evaluate the effect of stage factor (initial, middle, and final stage) on the behavioral (ball rotation time and number of errors) and quantitative changes in EMG activity (number of EMG on-phases and total and average EMG area) and EMG variability (CV of on-phase duration and area). Spearman’s rank correlation analysis was used to evaluate the relationship between the ball rotation time and CV of on-phase duration, and between the ball rotation time and CV of on-phase area for 30 trials for all participants. We also calculated the percent change in EMG activity and ball rotation time for both the variabilities as follows:

100-(averagevalueofthefinalstage/averagevalueoftheinitialstage)×100%

We then used Spearman’s rank correlation analysis to evaluate the correlation between the percent change of the variability in EMG activity and ball rotation time. In addition, we used Fisher’s Z-method to examine whether the strength of these correlation coefficients varied across muscles. Bonferroni’s correction was used for multiple comparisons.

## Results

### Behavioral changes

**Fig 3:**
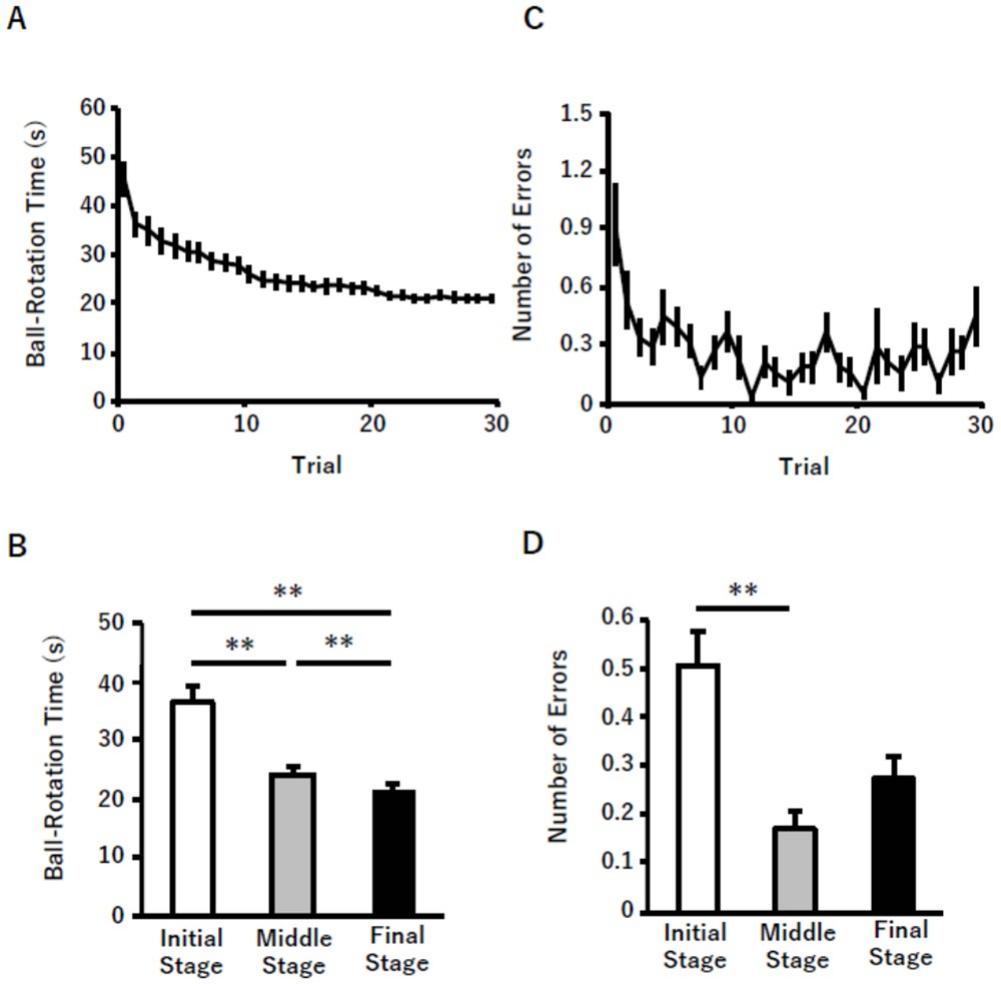
Results of behavioral changes. (A) Changes in the ball rotation time from trial 1 to 30. (B) Comparison of the mean (± SE) ball rotation time between the three stages (initial, trials 1–5; middle, trials 13–17; and final, trials 26–30) (C) Changes in number of errors from trial 1 to 30. (D) Comparison of the mean (± SE) number of errors between the three stages. (** p < 0.01, * p < 0.05).

The ball rotation time decreased with the number of completed trials (Fig 3A). Friedman’s test revealed a significant main effect of stage (initial, middle, and final) on the ball rotation time [χ ^2^ (2) = 39.5, p < 0.0005], with post-hoc multiple comparison tests confirming that the ball rotation time decreased in the order of initial, middle, and final stages (Fig 3B). Friedman’s test also revealed a significant main effect of stage on the number of errors [χ 2 (2) = 19.3, *p* < 0.0005, Fig 3C and 3D]. The post-hoc test revealed that the number of errors was significantly lower in the middle stage than in the initial stage. There were no significant differences in the number of errors between the initial and final stage and the middle and final stage.

### EMG changes

**Fig 4:**
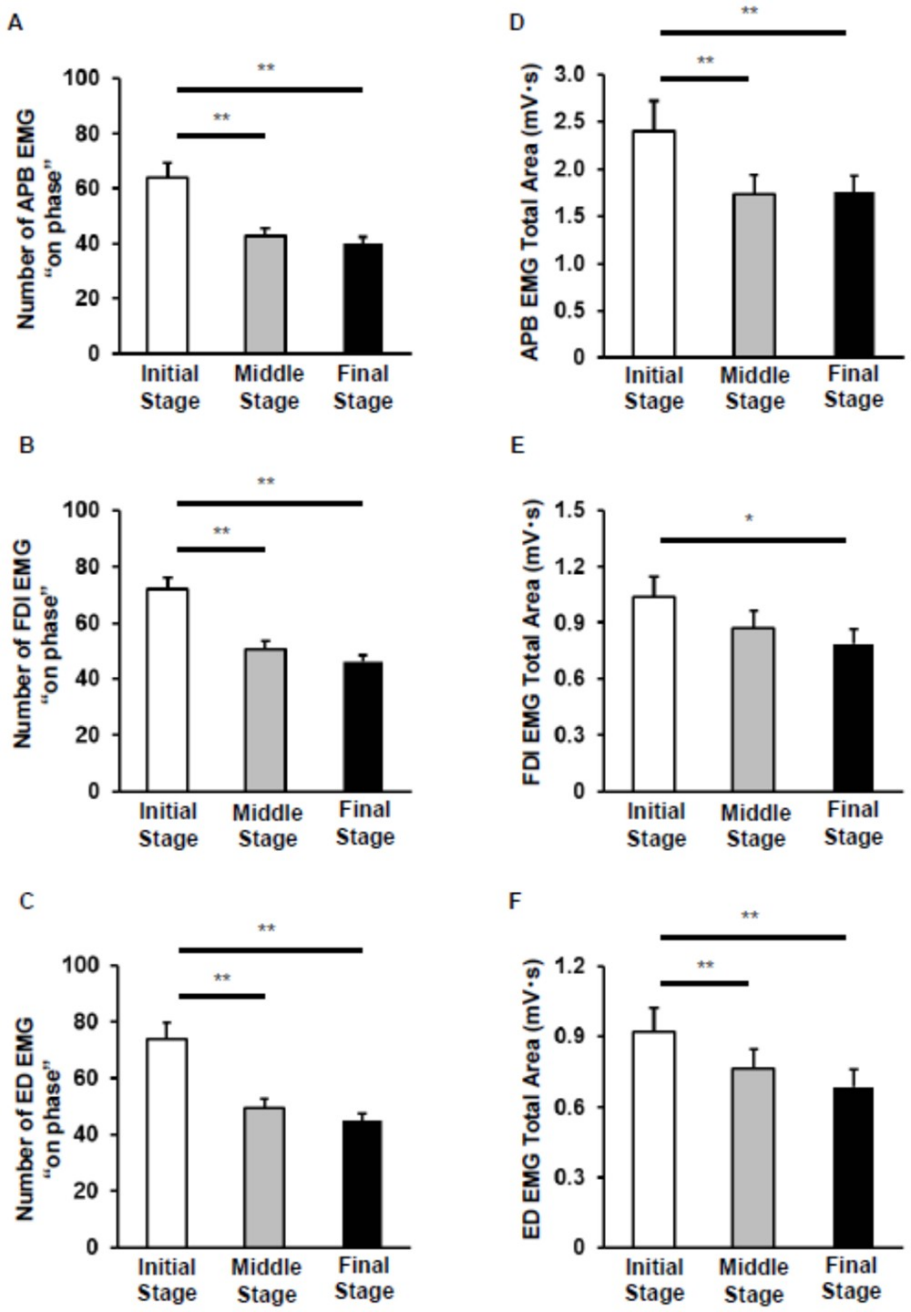
Changes in the number of electromyography (EMG) on-phases and total EMG area. (A-C) Comparison of the mean (± SE) number of EMG on-phases between the three stages (initial, trials 1–5; middle, trials 13–17; and final, trials 26–30) in the abductor pollicis brevis (APB) (A), first dorsal interosseous (FDI) (B), and extensor digitorum (ED) (C) muscles. (D–F) Differences in the mean (± SE) EMG total area of the APB, FDI and ED muscles between the three stages are shown in panels (D), (E), and (F), respectively. *n* = 37, 37, and 36 for APB, FDI, and ED, respectively (**; p < 0.01, *; p < 0.05).

One-way repeated ANOVA revealed a significant main effect of stage on the number of EMG on-phases for all the three muscles [APB: F (2, 72) = 37.8, p < 0.0005; FDI: F (2, 72) = 46.7, p < 0.0005; and ED: F (2, 70) = 29.8, p < 0.0005; Fig 4A–4C]. Post-hoc analysis confirmed that the number of on-phases decreased in the middle and final stages compared with that in the initial stage for all three muscles. Friedman’s test also revealed a significant main effect of stage on the total EMG area for each of the three muscles [APB: χ ^2^ (2) = 19.0, p < 0.0005; FDI: χ ^2^ (2) = 6.00, p = 0.0498; and ED: χ ^2^ (2) = 23.7 p = < 0.0005; Fig 4D–4F]. For the APB and ED muscles, the total EMG area was significantly decreased in the middle and final stages compared with that in the initial stage. For the FDI muscle, the total EMG area was significantly decreased in the final stage compared with that in the initial stage, and there were no significant differences between the initial and middle stage and between the middle and final stage. For the APB muscle, the average EMG area in the final stage was significantly larger than that in the initial and middle stages [APB: χ ^2^ (2) = 8.3, p < 0.016]. For the FDI muscle, the average EMG area increased in the final stage compared with that in the initial stage [FDI: χ ^2^ (2) = 13.7, p < 0.001]. However, for the ED muscle, there was no significant difference in the average EMG areas among the three stages [FDI: χ ^2^ (2) = 2.3, p = 0.32].

**Fig 5:**
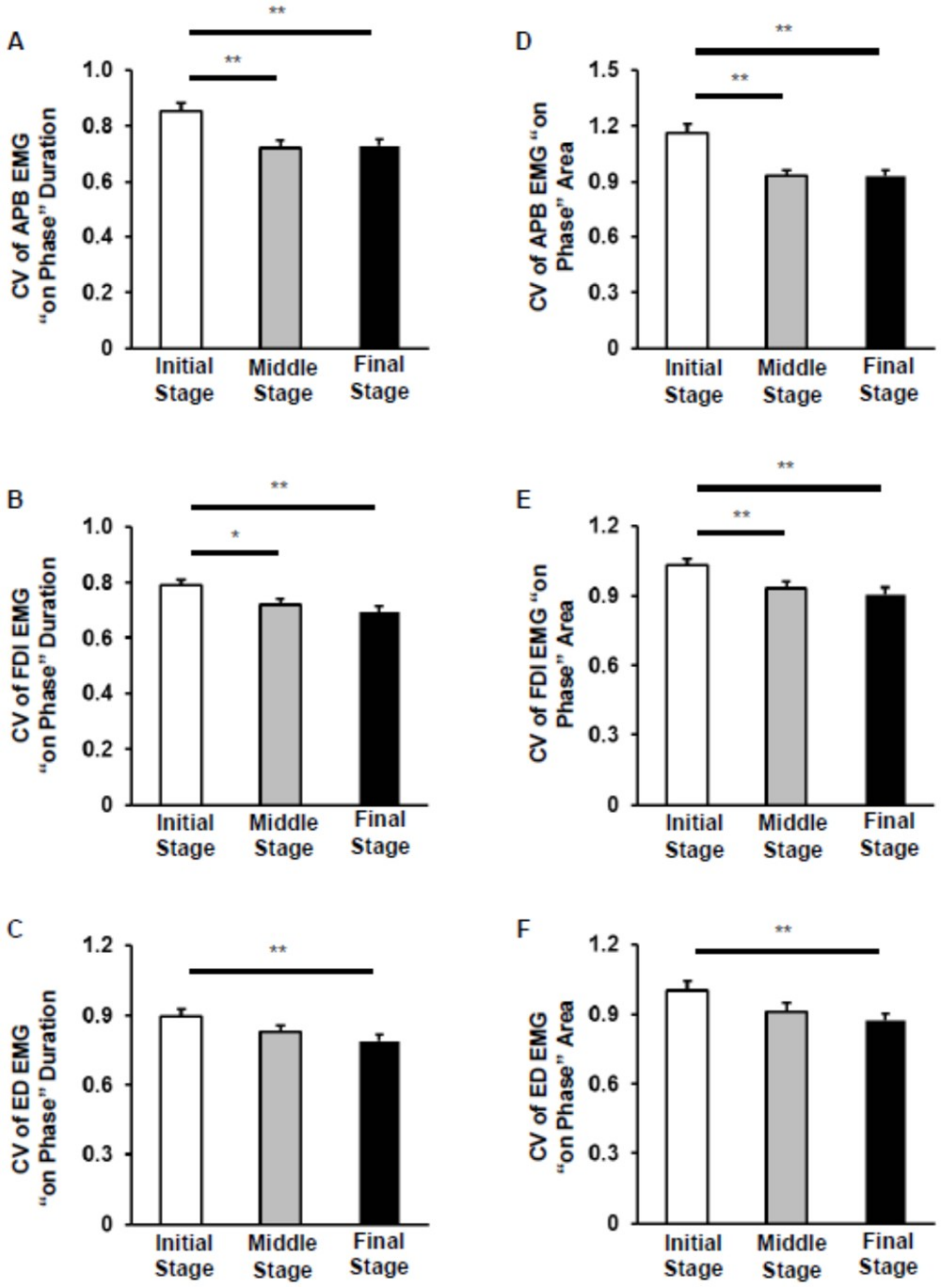
Changes in electromyography (EMG) variability. (A–C) Comparison of the mean (± SE) coefficient of variation (CV) of EMG on-phase duration between the three stages (initial, trials 1–5; middle, trials 13–17; and final, trials 26–30) in the abductor pollicis brevis (APB) (A), first dorsal interosseous (FDI) (B), and extensor digitorum (ED) (C) muscles. (D–F) The mean CV of EMG on-phase area for the APB, FDI, and ED muscles for the three stages are shown in panels (D), (E), and (F), respectively. *n* = 37, 37, and 36 for APB, FDI, and ED, respectively (** p < 0.01, * p < 0.05).

Friedman’s test revealed a significant main effect of stage on the CV of on-phase duration for all three muscles [APB: χ ^2^ (2) = 29.9, p < 0.0005; FDI: χ ^2^ (2) = 15.1, p = 0.001; and ED: χ ^2^ (2) = 11.5, p = 0.003; Fig 5A–5C]. For the APB and FDI muscles, the CV of on-phase duration was significantly decreased in the middle and final stages compared with that in the initial stage. In addition, it was significantly decreased in final stage compared with that in the initial stage for the ED muscle. Similarly, a significant main effect of stage on the CV of EMG on-phase area was noted for all three muscles [APB: χ ^2^ (2) = 38.5, p < 0.0005; FDI: χ ^2^ (2) = 16.4, p < 0.0005; and ED: χ ^2^ (2) = 11.1, p = 0.004; Fig 5D–5F]. The CV of EMG on-phase area for the APB and FDI muscles was significantly decreased in the middle and final stages compared with that in the initial stage. For the ED muscle, it was significantly decreased in the final stage compared with that in the initial stage.

### Relationship between motor skills and EMG variability

**Fig 6:**
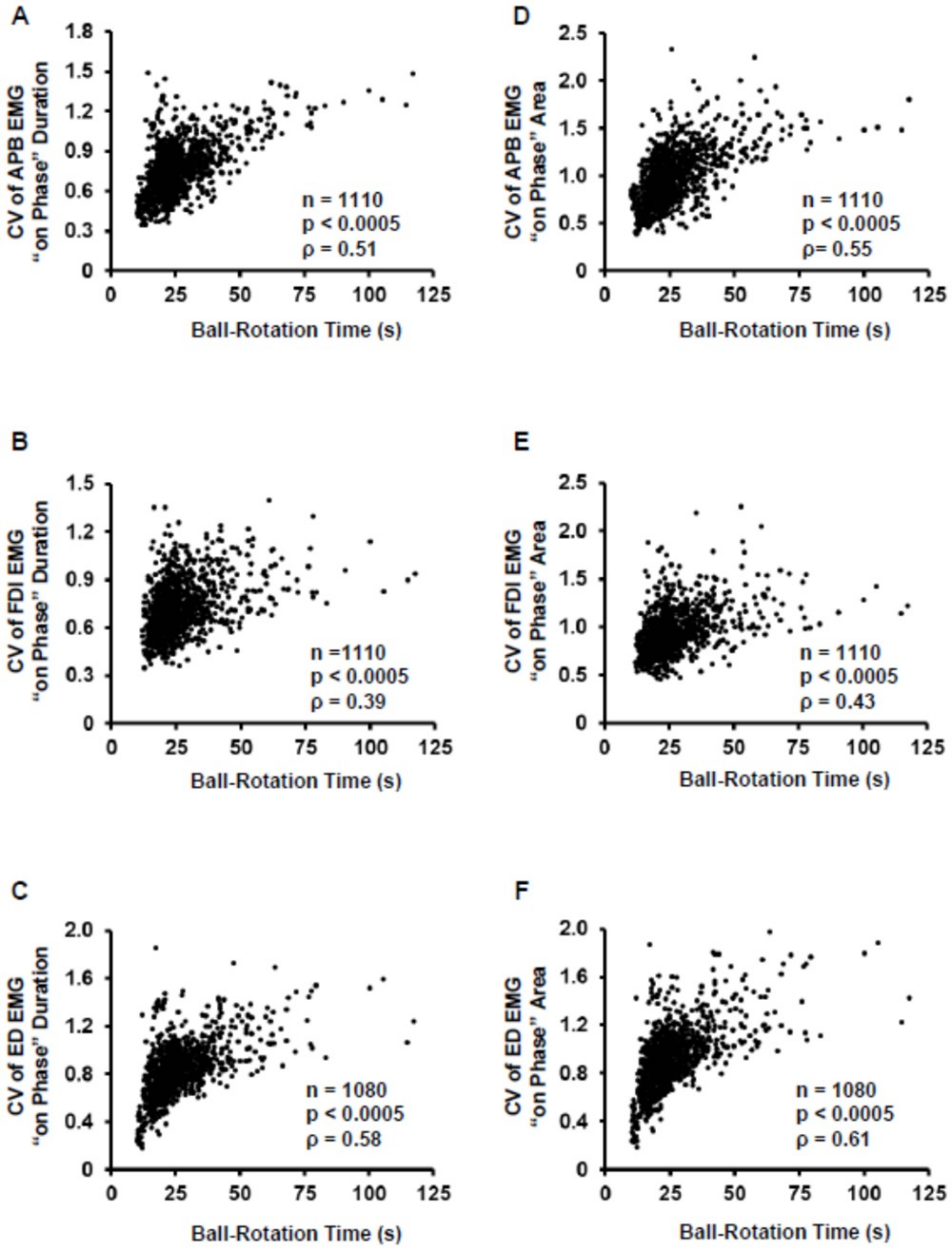
Correlations between ball rotation rime and electromyography (EMG) variability. (A–C) Spearman’s correlation coefficient between the ball rotation time and coefficient of variation (CV) of EMG on-phase duration for the abductor pollicis brevis (APB) (A), first dorsal interosseous (FDI) (B), and extensor digitorum (ED) (C) muscles. (D–F) Spearman’s correlation coefficient between the ball rotation time and CV of EMG on-phase area for the APB (D), FDI (E), and ED (F) muscles. Each closed circle represents the data for a single trial in one participant. The data for a total of 30 trials per participant are presented. *n* = 1110, 1110, and 1080 for APB, FDI, and ED, respectively.

Spearman’s rank correlation analysis showed significant correlations between the participants’ ball rotation time and the CV of on-phase duration for all three muscles (APB: ρ = 0.51, p < 0.0005; FDI: ρ = 0.39, p = < 0.0005; and ED: ρ = 0.58, p < 0.0005; Fig 6A–6C). The correlation coefficient between the ball rotation time and CV of on-phase duration was significantly smaller for the FDI muscle than for the APB (*z* = 3.65, *p* = 0.001) and ED (*z* = 5.76, *p* < 0.001) muscles. The ball rotation time was also significantly correlated with the CV of EMG on-phase area for all three muscles (APB: ρ = 0.55, p < 0.0005; FDI: ρ = 0.43, p < 0.0005; and ED: ρ = 0.61, p < 0.0005; Fig 6D–6F). The correlation coefficient between the ball rotation time and CV of on-phase area was significantly smaller for the FDI muscle than for the APB (*z* = 3.70, *p* = 0.001) and ED (*z* = 5.72, *p* < 0.001) muscles.

**Fig 7:**
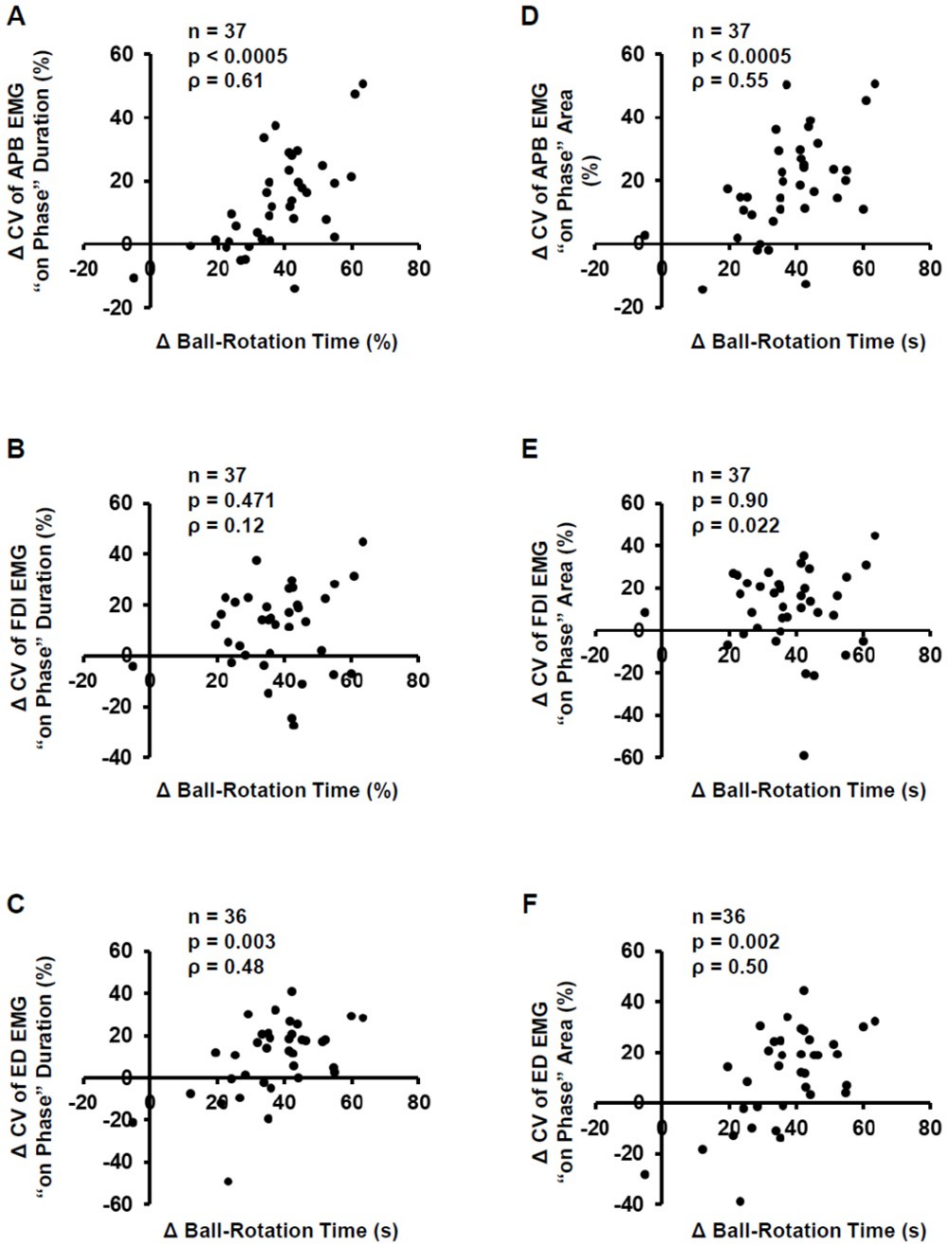
Correlations between the improvements in ball rotation time and reduction in electromyography (EMG) variability. (A–C) Spearman’s correlation coefficient between the percent change (Δ) in ball rotation time and coefficient of variation (ΔCV) of EMG on-phase duration in abductor pollicis brevis (APB) (A), first dorsal interosseous (FDI) (B), and extensor digitorum (ED) (C) muscles. (D–F) Spearman’s correlation coefficient between Δ ball rotation time and ΔCV of EMG on-phase area in the APB (D), FDI (E) and ED (F) muscles.

There were significant correlations between the percent change in ball rotation time and the CV of on-phase duration for the APB and ED muscles but not for the FDI muscle (APB: ρ = 0.61, p < 0.0005; FDI: ρ = 0.12, p = 0.471; and ED: ρ = 0.48, p = 0.003; Fig 7A–7C). The correlation coefficient between the percent change in ball rotation time and CV of on-phase duration was significantly smaller for the FDI muscle than for the APB muscle (*z* = 2.41, *p* = 0.048). Similarly, there were significant correlations between the percent change in ball rotation time and CV of EMG on-phase area for the APB and ED muscles but not for the FDI muscle (APB: ρ = 0.55, p < 0.0005; FDI: ρ = 0.02, p = 0.90; and ED: ρ = 0.50, p = 0.002; Fig 7D–7F). The correlation coefficient between the percent change in ball rotation time and CV of the on-phase area was significantly smaller for the FDI muscle than for the APB muscle (*z* = 2.46, *p* = 0.042).

## Discussion

The results of this study demonstrated that the participants’ ball rotation time was significantly shortened by performing the ball rotation task repeatedly. These improvements in motor skills were accompanied by significant decreases in the variability in EMG on-phase duration and area. These results suggest that repeatedly practicing a dexterous multi-finger movement reduces temporal and quantitative EMG variability.

Moore and Marteniuk [14] reported that repeatedly practicing a task that involves a 45° elbow extension in the horizontal plane reduces the EMG variability with a kinematic improvement in motor performance. Gabriel et al. [15] investigated how kinematics and EMG variability change during the process of learning an 80° elbow flexion task and reported that variability in the EMG amplitude in the agonist muscle and that in motor time in both the agonist and antagonist muscles decrease with kinematic improvement. The results of the present study were consistent with these results. However, in daily life and sports activities, single-joint movements investigated in the previous two studies are seldom used, and complex joint movements using multiple muscles are required. Therefore, it is worth noting that, as shown in the present study, repetitive practice gradually reduces temporal and quantitative variability in muscle activity even for movements that require coordinated multi-finger muscle activity, similar to the previous findings for single-joint movements.

Another finding of the present study was that the percent change in ball rotation time significantly correlated with the percent change in CV of EMG on-phase duration and EMG area for the APB and ED muscles, suggesting a reduction in the variability in EMG activity is closely related to improvement in motor skills. Conversely, there was no observed similar significant correlation for the FDI muscle. Furthermore, the correlation coefficients between percent change in ball rotation time and percent change in CV of on-phase duration and area were significantly smaller for the FDI muscle than for the APB muscle. These differences are reasonable given the functional roles of each muscle in the task. The ball rotation task was usually performed for abduction–adduction movements of the thumb and for flexion–extension movements of the four fingers; therefore, the APB muscle, an agonist of thumb abduction, and the ED muscle, an agonist of finger extension, would contribute strongly to this movement. Conversely, the FDI muscle is an agonist of the abduction of the index finger; therefore, it would not be strongly involved in ball rotation movement. As a result, differences in the results for these muscles may reflect their specific contributions to the ball rotation task. These assumptions are also supported by our results that the correlation coefficients between ball rotation time and CV of on-phase duration and area were significantly smaller for the FDI muscle than for the APB and ED muscles.

Correlation analysis also showed that the individual participants’ motor skills strongly correlated with the variability in EMG activity, particularly for the APB and ED muscles, suggesting that the variability in EMG activity of a muscle that is strongly involved in a movement is an indicator of the individual’s level of motor skills. Previous studies that have investigated the relationship between motor skills and EMG activity have reported differences in muscle activity between experts and non-experts and between athletes and non-athletes, including differences in the EMG amplitude [19], EMG power spectrum [20], co-contraction patterns of agonist and antagonist muscles [21, 22], and variability in the EMG activation time [21]. However, to the best of our knowledge, no study has reported a correlation between individuals’ motor skills and muscle activity patterns; therefore, we believe that the findings of the present study provide novel insights regarding the cause of individual differences in motor skills.

Although there was an increase in the average EMG areas for the APB and FDI muscles, the number of on-phases and the total EMG areas for all three muscles decreased significantly via the motor learning process. These results suggest that a decrease in the number of on-phases contributes to total EMG area reduction. We speculate that muscle activity, which was observed in the initial stage when there was no need to be active, decreases as the motor learning process progresses. Such optimization of muscle electrical activity during the motor learning process is consistent with the findings of previous studies. Gribble et al. [23] reported the considerable co-contraction of multiple muscles during the early stage of motor learning that decreases as motor learning progresses. Co-contraction of muscles plays a role in reducing performance errors by enhancing joint viscoelasticity [24, 25]. It is thought that the co-contraction of muscles decreases gradually as individuals develop an internal model of the movement through motor learning [24, 26–28]. We speculate that the acquisition of such internal models with motor learning is related not only to the reduction in unnecessary muscle activity but also to the reduction in the temporal and quantitative variability in EMG activity, similar to the findings observed in the present study. However, further research is warranted to confirm this hypothesis.

One important limitation of this study is that the experimental design used in this study investigated short-term learning effects. Therefore, whether the changes in EMG variability obtained in this study are similarly observed in long-term learning is not clear. Future work is needed to determine whether changes in the variability of EMG activity differ between long-term and short-term learning.

## Conclusions

The results of this study demonstrated that temporal and quantitative variability in EMG activity decreases with the progress of motor learning and that the extent of decrease is positively correlated with the improvement in motor skills. These novel findings provide important insights into the relationship between variability in muscle electrical activity and learning of fine motor skills. Furthermore, the finding that variability in EMG activity is closely related to individual motor skills suggests that variability in EMG activity should be a beneficial indicator for the evaluation of the degree of an individual’s motor skills.

## Supporting information

S1 Table Changes in ball rotation time and number of errors for individual participants. (XLSX)

S2 Table Changes in APB EMG activity for individual participants. (XLSX)

S3 Table Changes in FDI EMG activity for individual participants. (XLSX)

S4 Table Changes in ED EMG activity for individual participants. (XLSX)

S5 Table Summary of the results. (XLSX)

## References

[1] Vorro J, Hobart D. Kinematic and myoelectric analysis of skill acquisition: II. 150cm subject group. Arch Phys Med Rehabil. 1981;62(11):582–9. 7316716

[2] Ludwig DA. EMG changes during acquisition of a motor skill. Am J Phys Med. 1982;61(5):229–43. 7124913

[3] Lay BS, Sparrow WA, Hughes KM, O’Dwyer NJ. Practice effects on coordination and control, metabolic energy expenditure, and muscle activation. Human Movement Science. 2002;21(5–6):807–30. 10.1016/s0167-9457(02)00166-5 12620721

[4] Liang N, Yamashita T, Ni Z, Takahashi M, Murakami T, Yahagi S, et al Temporal modulations of agonist and antagonist muscle activities accompanying improved performance of ballistic movements. Hum Mov Sci. 2008;27(1):12–28. 10.1016/j.humov.2007.05.007 17936390

[5] Zhang H, Kumar A, Luo X, Svensson K, Trulsson M, Svensson P. Effect of short-term training on fine motor control in trigeminally innervated versus spinally innervated muscles. Hum Mov Sci. 2018;58:132–9. 10.1016/j.humov.2018.01.013 29426038

[6] Brueckner D, Gopfert B, Kiss R, Muehlbauer T. Effects of motor practice on learning a dynamic balance task in healthy young adults: A wavelet-based time-frequency analysis. Gait Posture. 2019;70:264–9. 10.1016/j.gaitpost.2019.03.019 30909006

[7] Thomas E, French R, Alizee G, Coull JT. Having your cake and eating it: Faster responses with reduced muscular activation while learning a temporal interval. Neuroscience. 2019;410:68–75. 10.1016/j.neuroscience.2019.05.003 31082534

[8] Sawers A, Allen JL, Ting LH. Long-term training modifies the modular structure and organization of walking balance control. J Neurophysiol. 2015;114(6):3359–73. 10.1152/jn.00758.2015 26467521 PMC4868379

[9] Winter DA, Yack HJ. EMG profiles during normal human walking: stride-to-stride and inter-subject variability. Electroencephalogr Clin Neurophysiol. 1987;67(5):402–11. 10.1016/0013-4694(87)90003-4 2444408

[10] Guidetti L, Rivellini G, Figura F. EMG patterns during running: Intra- and inter-individual variability. J Electromyogr Kinesiol. 1996;6(1):37–48. 10.1016/1050-6411(95)00015-1 20719661

[11] Martens J, Daly D, Deschamps K, Fernandes RJ, Staes F. Intra-Individual Variability of Surface Electromyography in Front Crawl Swimming. PLoS One. 2015;10(12):e0144998 10.1371/journal.pone.0144998 26673163 PMC4682934

[12] Granata KP, Padua DA, Abel MF. Repeatability of surface EMG during gait in children. Gait Posture. 2005;22(4):346–50. 10.1016/j.gaitpost.2004.11.014 16274917 PMC1628350

[13] Tirosh O, Sangeux M, Wong M, Thomason P, Graham HK. Walking speed effects on the lower limb electromyographic variability of healthy children aged 7–16 years. J Electromyogr Kinesiol. 2013;23(6):1451–9. 10.1016/j.jelekin.2013.06.002 23886484

[14] Moore SP, Marteniuk RG. Kinematic and electromyographic changes that occur as a function of learning a time-constrained aiming task. J Mot Behav. 1986;18(4):397–426. 10.1080/00222895.1986.10735388 15138139

[15] Gabriel DA. Changes in kinematic and EMG variability while practicing a maximal performance task. J Electromyogr Kinesiol. 2002;12(5):407–12. 10.1016/s1050-6411(02)00026-3 12223174

[16] Matsumura M, Sadato N, Kochiyama T, Nakamura S, Naito E, Matsunami K, et al Role of the cerebellum in implicit motor skill learning: a PET study. Brain Res Bull. 2004;63(6):471–83. 10.1016/j.brainresbull.2004.04.008 15249112

[17] Uehara S, Nambu I, Tomatsu S, Lee J, Kakei S, Naito E. Improving human plateaued motor skill with somatic stimulation. PLoS One. 2011;6(10):e25670 10.1371/journal.pone.0025670 21991331 PMC3186792

[18] Oldfield RC. The assessment and analysis of handedness: the Edinburgh inventory. Neuropsychologia. 1971;9(1):97–113. 10.1016/0028-3932(71)90067-4 5146491

[19] Furuya S, Osu R, Kinoshita H. Effective utilization of gravity during arm downswing in keystrokes by expert pianists. Neuroscience. 2009;164(2):822–31. 10.1016/j.neuroscience.2009.08.024 19698766

[20] Saito S, Obata H, Kuno-Mizumura M, Nakazawa K. On the skilled plantar flexor motor action and unique electromyographic activity of ballet dancers. Exp Brain Res. 2018;236(2):355–64. 10.1007/s00221-017-5131-0 29147730

[21] Fujii S, Kudo K, Shinya M, Ohtsuki T, Oda S. Wrist muscle activity during rapid unimanual tapping with a drumstick in drummers and nondrummers. Motor Control. 2009;13(3):237–50. 10.1123/mcj.13.3.237 19799164

[22] Kim M, Kim Y, Kim H, Yoon B. Specific muscle synergies in national elite female ice hockey players in response to unexpected external perturbation. J Sports Sci. 2018;36(3):319–25. 10.1080/02640414.2017.1306090 28415899

[23] Gribble PL, Mullin LI, Cothros N, Mattar A. Role of cocontraction in arm movement accuracy. J Neurophysiol. 2003;89(5):2396–405. 10.1152/jn.01020.2002 12611935

[24] Osu R, Franklin DW, Kato H, Gomi H, Domen K, Yoshioka T, et al Short- and long-term changes in joint co-contraction associated with motor learning as revealed from surface EMG. J Neurophysiol. 2002;88(2):991–1004. 10.1152/jn.2002.88.2.991 12163548

[25] Heald JB, Franklin DW, Wolpert DM. Increasing muscle co-contraction speeds up internal model acquisition during dynamic motor learning. Sci Rep. 2018;8(1):16355 10.1038/s41598-018-34737-5 30397273 PMC6218508

[26] Thoroughman KA, Shadmehr R. Electromyographic correlates of learning an internal model of reaching movements. J Neurosci. 1999;19(19):8573–88. 10.1523/JNEUROSCI.19-19-08573.1999 10493757 PMC6783008

[27] Franklin DW, Osu R, Burdet E, Kawato M, Milner TE. Adaptation to stable and unstable dynamics achieved by combined impedance control and inverse dynamics model. J Neurophysiol. 2003;90(5):3270–82. 10.1152/jn.01112.2002 14615432

[28] Franklin S, Wolpert DM, Franklin DW. Visuomotor feedback gains upregulate during the learning of novel dynamics. J Neurophysiol. 2012;108(2):467–78. 10.1152/jn.01123.2011 22539828 PMC3404796

